# Exercise-Induced Neuroplasticity in Parkinson's Disease: A Metasynthesis of the Literature

**DOI:** 10.1155/2020/8961493

**Published:** 2020-03-05

**Authors:** Hanna Johansson, Maria Hagströmer, Wilhelmus J. A. Grooten, Erika Franzén

**Affiliations:** ^1^Department of Neurobiology, Care Sciences and Society, Division of Physiotherapy, Karolinska Institutet, Stockholm, Sweden; ^2^Function Area Occupational Therapy & Physiotherapy, Allied Health Professionals Function, Karolinska University Hospital, Stockholm, Sweden; ^3^Department of Health Promoting Science, Sophiahemmet University, Stockholm, Sweden; ^4^Stockholms Sjukhem Foundation, Stockholm, Sweden

## Abstract

Parkinson's disease (PD) is a neurodegenerative disorder for which there is currently only symptomatic treatment. During the last decade, there has been an increased interest in investigating physical exercise as a neuroprotective mechanism in PD. Animal studies have suggested that exercise may in fact induce neuroplastic changes, but evidence in humans is still scarce. A handful of reviews have previously reported on exercise-induced neuroplasticity in humans with PD, but few have been systematic, or have mixed studies on both animals and humans, or focused on one neuroplastic outcome only. Here, we provide a systematic review and metasynthesis of the published studies on humans in this research field where we have also included different methods of evaluating neuroplasticity. Our results indicate that various forms of physical exercise may lead to changes in various markers of neuroplasticity. A narrative synthesis suggests that brain function and structure can be altered in a positive direction after an exercise period, whereas a meta-analysis on neurochemical adaptations after exercise points in disparate directions. Finally, a GRADE analysis showed that the current overall level of evidence for exercise-induced neuroplasticity in people with PD is very low. Our results demonstrate that even though the results in this area point in a positive direction, researchers need to provide studies of higher quality using more rigorous methodology.

## 1. Introduction

Parkinson's disease (PD) is a neurodegenerative disorder for which there is no curative treatment today. Prevalence differs according to age, sex, and geographic location, but an overall worldwide estimate is 315 per 100 000 people [1]. The histopathology of PD is classically characterized by a loss of dopaminergic neurons in the substantia nigra, and the cardinal features of PD include resting tremor, rigidity, and bradykinesia. As the disease progresses, postural instability and gait disturbances also become more severe. Apart from these aforementioned symptoms, people with PD are also affected by various nonmotor features such as sleep disorders, psychiatric symptoms, and cognitive dysfunction [2].

There is a growing body of research highlighting the role of physical exercise as an essential part of managing PD, by means of neuroprotective mechanisms [3, 4]. Neuroplasticity can be defined as the capability of the central nervous system to adapt itself in response to internal and external stimuli. In short, it is the way that neurons alter their structure and function to cope with their environment [5]. There are several different techniques of evaluating neuroplasticity, such as brain imaging and sampling blood or cerebrospinal fluid in order to investigate nerve growth factors. Some of the methods measure neurochemical processes and others measure brain function and/or brain structures, but the choice of analysis needs to be regulated by the research question at hand. The quantitatively synthesized and systematically graded evidence on exercise-induced neuroplasticity in neurological populations to date is however scarce. A meta-analysis from 2017 suggests that a period of regular aerobic exercise increases the level of brain-derived neurotrophic factor (BDNF) in a combined sample of studies on stroke, Multiple Sclerosis, and PD [6]. Animal studies suggest that exercise may induce neuroplastic changes in PD [7–10], but only a few studies have been conducted on humans with PD. A handful of reviews have reported on this topic previously [3, 4, 11–13], but only one was conducted and reported in a systematic manner [4]. Further, they either included both human and animal studies [3, 11–13] or focused on one neuroplastic outcome only [4].

Understanding if and how physical exercise mediates changes in neuroplasticity could help guide the development of neurorehabilitation by focusing on therapies that maximize neural plasticity. There is a need to perform an updated synthesis of the literature on this topic, in order to establish the current evidence. The objective of this systematic review and metasynthesis is therefore to establish the current evidence on postintervention effects of a period of physical exercise on neuroplasticity in people with idiopathic PD.

## 2. Method

The design was a systematic review and metasynthesis. A review protocol was established and registered in PROSPERO (ID CRD42017057834).

### 2.1. Study Selection

Exhaustive searches were conducted by librarians after consultations with two of the review authors. Relevant articles were identified through electronic searches in the following databases: Medline (Ovid), Embase, Cinahl (EbscoHost), and PEDro. Intervention studies on humans with idiopathic PD were included. Regarding the intervention, studies where the intervention was *any type* of physical exercise performed repeatedly (i.e., not just on one occasion), or where the intervention was a combination of physical exercise and mental training, but where the physical exercise made up the majority of the intervention were included. There was no exclusion based on the disease stage, age, gender, or medication, or for publication date or language. Study exclusion criteria were nonidiopathic PD, studies examining only acute (<24 hours) effects of exercise, or studies with a combination of physical exercise and mental training, where the mental training made up the main part of the intervention. For details on search strategy and information (see Supplementary Material (SM) ([Supplementary-material supplementary-material-1])).

Studies identified through database searches were screened by two review authors (HJ and EF) blinded to each other's decisions using the web-based tool *Rayyan* [14], on the basis of title and abstract. Studies were excluded when it was clear from the article title or abstract that the trial was not relevant or if it did not meet the inclusion criteria. After the initial screening, the two review authors unblinded their decisions, and disagreements were resolved through discussions with a third review author (MH). Reference lists of all included studies were screened for eligible studies.

### 2.2. Critical Appraisal Method

A modified version of the 27 item Downs and Black checklist was selected in order to assess research quality of the included studies. The checklist comprises an overall quality index and four subscales: reporting, external quality, internal validity bias, and internal validity confounding. [15] For the purpose of this review, item 27 was collapsed into a yes (1) or no (0) question, with yes meaning that a power calculation was reported and no subsequently meaning that authors did not provide a power calculation. The modified version thereby had a maximum score of 28, and the following overall quality index grades were employed as suggested by O'Connor et al. [16]: “excellent” (24–28 points), “good” (19–23 points), “fair” (14–18 points), or “poor” (<14 points).

### 2.3. Data Extraction and Data Synthesis

First, predefined details of data from the studies were inserted into a coding sheet. This served as a broad map to screen for potential commonalities and diversities between the studies [17]. The included studies were then grouped into three outcome domains based on methods used to measure neuroplasticity: *neurochemical*, *brain function*, or *brain structure.*

The following data were retrieved from each study: (1) data on design, setting, recruitment process, and inclusion and exclusion criteria based on age, Hoehn and Yahr stage [18], and cognition were. Only participants with PD were included in the review, and reported designs were therefore reassessed without including healthy controls and subsequently revised accordingly. In the study conducted by Maidan et al., it was decided that the control group would be considered the intervention group in this review, as we were interested in the physical exercise as opposed to the virtual reality (VR) component [19]. (2) Outcome measures used; (3) information on exercise type and intensity according to duration (number of weeks), frequency (sessions per week), and length of exercise (minutes per session); (4) sample characteristics regarding size, age, and Hoehn and Yahr stage; and (5) values (*p* values, confidence intervals, and effect sizes) and/or descriptives on posttraining result regarding neuroplasticity as well as other outcomes.

Mean averages of participant characteristics (*n*) and intervention intensity (duration, frequency, and length) were calculated. Mean average and standard deviation of age of participants were weighted based on sample size. Studies where the aforementioned variables were not reported were not included in the pooled calculations.

A narrative synthesis was performed in which the direction (positive, negative, or absence) of change in each method used to measure neuroplasticity was stated. Lastly, a quantitative synthesis was conducted when at least two studies within the same outcome domain provided aggregable and comparable outcome data. Values of means and standard deviations of pre- and posttraining intervention were entered into the Metaessentials workbook 4 (differences between dependent groups—continuous data.xlsx), where effect sizes were generated using a random effects model [20]. As no *r* values were provided in any of the meta-analyzed studies, all three studies were assigned the same *r* value. The analysis was then repeated with different correlation coefficients: 0.25 (poor), 0.60 (moderate), and 0.85 (very strong) [21]. The assumption that the studies could be assigned the same *r* value was based on the belief that the outcome (BDNF) would have been relatively stable during the intervention period (4–8 weeks) in a group of people with mild to moderate PD who did not receive any intervention.

### 2.4. Evidence Synthesis

The GRADE method was used to assess the overall level of evidence (LoE) on whether exercise can induce neuroplasticity in people with PD [22]. The initial LoE was set based on the judgement of *study phase*. After this, judgement of the following factors could downgrade the LoE: *study limitations*, *inconsistency*, *indirectness*, *imprecision*, *and publication bias*. Finally, an overall 4-LoE was set: ++++ (high), +++ (moderate), ++ (low), or + (very low). [23]

## 3. Results

### 3.1. Study Selection

**Figure 1:**
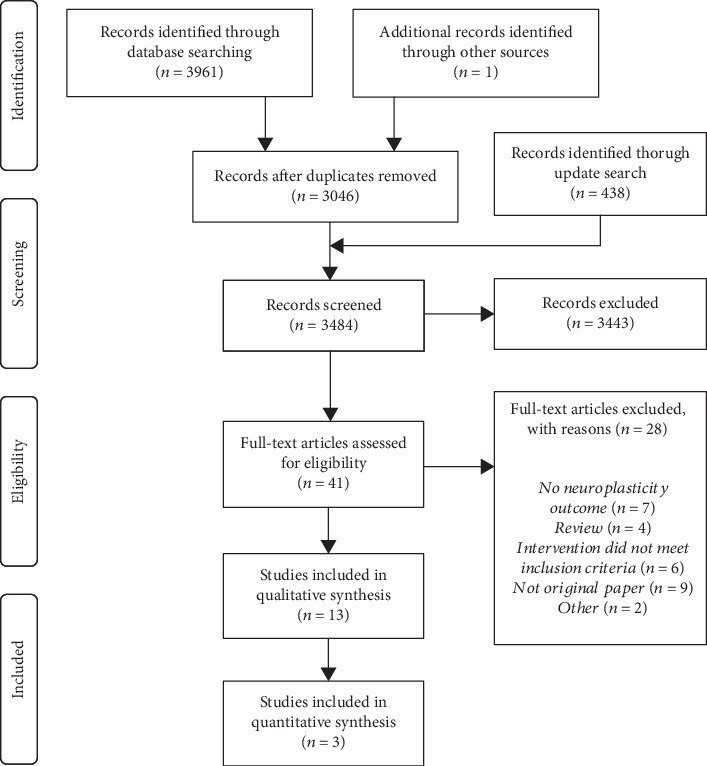
PRISMA flow diagram modified from Moher D, Liberati A, Tetzlaff J, Altman DG, The PRISMA Group (2009). Preferred Reporting Items for Systematic Reviews and Meta-Analyses: The PRISMA Statement. PLoS Med 6 [7].

The initial database search (February 2017) and an update search (November 2017) yielded a total of 3484 abstracts after duplicates were removed. After initial screening of these abstracts, 3443 were excluded based on the aforementioned criteria, leaving a total of 41 articles for further evaluation in full length. Out of these articles, 28 were excluded (see SM for reasons), leaving a total of 13 to be included in the qualitative synthesis. There was disagreement regarding one article, and this article was therefore decided upon in collaboration with the third review author (MH) (see Figure 1 for a description of the screening process presented with a PRISMA Flow Diagram [24]).

### 3.2. Description of the Studies Included in the Analysis

#### 3.2.1. Design Characteristics

The studies were conducted in various countries (Brazil, Canada, Germany, Israel, Italy, Spain, and USA) and settings (three inpatient and ten outpatient). Two of the studies from the USA had the same first author [25, 26], and two of the studies from Italy had three overlapping authors, either as first author or as coauthor [27, 28]. Three studies included specific age intervals (60–90 years, 45–80 years, and 30–65 years, respectively) [19, 29, 30], whereas the rest did not exclude based on age. Six studies included participants according to disease stage based on the Hoehn and Yahr scale [25, 28–32] (see Table 1 for study characteristics).

#### 3.2.2. Sample Characteristics

Total sample sizes were small, ranging from 1–34 participants, rendering a total of 151 intervention group participants and 63 controls for this review. Intervention group samples ranged from 1–20 participants (mean 11.6, SD 6.1), and control group samples from 2–17 (mean 10.5, SD 5.1). The pooled mean age of intervention group participants was 64.6 years (pooled SD 7.6) and 64.2 years (pooled SD 7.6) among control group participants (see Table 1 for sample characteristics for each study).

#### 3.2.3. Outcome Measures of Neuroplasticity

Seven different methods were used to measure neuroplasticity, and these were further operationalized into three domains: *neurochemical* (level of brain-derived neurotrophic factor (BDNF) in blood or serum (three studies) [28, 31, 33] and BDNF-TrkB signaling (one study)) [27], *brain function* (functional MRI (fMRI) (four studies) [19, 32, 34, 35], electroencephalogram (EEG) (one study) [29], positron emission tomography (PET) (two studies) [26, 36], transcranial magnetic stimulation (TMS) (one study)) [25], and *brain structure* (magnetic resonance imaging (MRI) (one study)) [34]. Assessment details are summarized in Table 1.

#### 3.2.4. Intervention and Behavior

With regard to intervention and control groups, several different types of physical exercise were employed (see Table 2 for details). The mean number of weeks per training period was 6.5 (SD 3.2), ranging from one to twelve; the mean number of training sessions per week was 5.8 (SD 5.3), ranging from one to fifteen; and the mean number of minutes per session was 56.1 (SD 7.4), ranging from 40 to 60. Three studies either did not conduct behavioral assessments [30, 35] or did not report behavior for the investigated subsample [25]. The other studies all showed improvements in various behavioral outcomes after the exercise period (see Table 2 for information).

### 3.3. Study Quality

Overall quality index score of the modified Downs and Black checklist ranged from 6 to 20 points, with a median of 14 points. A majority (nine) of the studies were graded as having “fair” quality, three studies were graded as having “poor” quality, and one as having “good” quality (see SM). Index scores are also stated in Table 3.

### 3.4. Narrative Synthesis

Three studies using blood sampling methods [27, 28, 33] showed positive results on neuroplasticity after a period of physical exercise. However, a fourth study within the neurochemical domain, Angelucci et al., showed no effects [31]. In the seven studies in which brain function was the main outcome measure, five different methods showed positive effects on neuroplasticity. For the outcome brain structure, only one study was found, Sehm et al. [34], which showed positive effects of exercise on neuroplasticity. All in all, the narrative results showed a clear effect of exercise on neuroplasticity across the outcomes brain function and brain structure, but unclear results were found in the neurochemical domain. The narrative syntheses are summarized in Table 3.

### 3.5. Quantitative Synthesis

#### 3.5.1. Neurochemical

**Figure 2:**
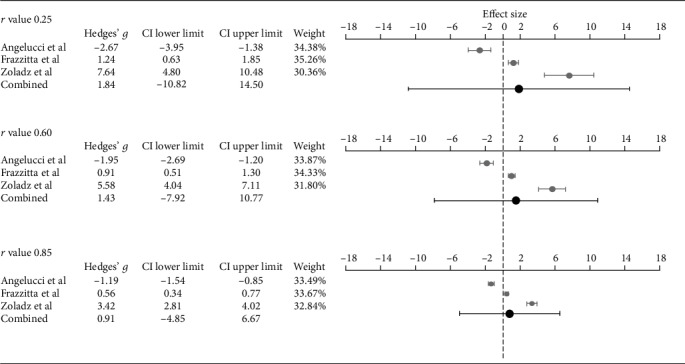
Meta-analyses and forest plots of included studies using three different *r* values, showing effect sizes (Hedges' *g*) of change in levels of brain-derived neurotrophic factor from pre- to postintervention.

Change in neurochemical biomarkers from pre- to post-training was measured in four studies [27, 28, 31, 33], including a total of 61 participants. Three studies provided aggregable and continuous data for inclusion in a meta-analysis [28, 31, 33]. Two of these studies did not have a control group and therefore the control group from the third study was removed for the meta-analysis; hence, they were treated as dependent groups. One study did not provide absolute pre- and post-values [31], so approximate values of means and SD's were calculated from measuring the included graph in an enlarged format. Error in measurement was controlled for by repeating the analysis with values close in range. Values of BDNF levels were converted to the same unit (ng/mL) for all studies. (1 gram = 1 000 000 000 nanogram) See Figure 2 for meta-analyses and forest plots. Results of the meta-analyses show that the overall effect size was small and ranged between 0.91 and 1.84, dependent on the choice of correlation coefficient. The confidence interval of the combined effect size includes zero in all three scenarios, indicating that the overall effect is nonsignificant. When looking at heterogeneity, *p* values of all three meta-analyses are <0.001 indicating a degree of heterogeneity among the studies. This is further supported by their respective *I*^2^ values, all being above 96% which suggests that the studies cannot be considered to be of the same population.

### 3.6. Brain Function

Change in brain function from pre- to post-training was measured in eight studies [19, 25, 26, 29, 30, 32, 35, 36], including a total of 132 participants. Given the heterogenic nature of measurement methods, no meta-analysis could be performed within this domain.

### 3.7. Brain Structure

Change in brain structure from pre- to post-training was measured in one study [34], conducted on 20 participants with PD (another 16 healthy controls were excluded from this review). Given that there was only one study, no meta-analysis could be conducted.

### 3.8. Overall Evidence Synthesis

Based on the GRADE synthesis, the results showed very low level of evidence that a period of physical exercise induces neuroplasticity in people with PD. Downgrading was due to “study limitations” and “imprecision”; see Table 4.

## 4. Discussion

### 4.1. Summary of Evidence

The objective was to establish the current evidence for exercise-induced neuroplasticity in people with idiopathic PD, and the results indicate that various forms of physical exercise may lead to changes in a range of markers of neuroplasticity. The narrative synthesis suggests that both brain function and structure can be altered in a positive direction after an exercise period. However, studies on neurochemical adaptations after exercise point in disparate directions, with some studies showing an increase after training, while others report unchanged values from baseline to post-intervention. Finally, a concerted GRADE analysis showed that the overall level of evidence for exercise-induced neuroplasticity in people with PD as of today is very low.

To our knowledge, only one other published review has shown meta-analyzed results of BDNF values before and after training in a PD-specific sample [4]. Their meta-analysis showed a significant summary effect size in favor of the experimental group, but the methods used differ from ours on several aspects. Firstly, we excluded studies where the intervention was not primarily focused on physical exercise; hence, the study by Sajatovic et al. [37] was not included. Secondly, our meta-analysis was conducted without control groups (given that only one of the studies were of RCT design). Even though we repeated the meta-analysis three times using different levels of correlation, the CI of the combined effect size remained nonsignificant. Although making assumptions about *r* values instead of using the correct ones can be considered a limitation to our methodology, the results remain nonsignificant, independently of the level of correlation, indicating the robustness of the results.

Within the outcome domains brain function and structure, our narrative syntheses present more consistent results pointing to the positive effect of exercise on the brains ability to adapt and restructure in people with PD. These results should however be interpreted with caution given the limited number and low methodological quality of the included studies and the inability to quantitatively synthesize them. There was an overall, severe underreporting of effect sizes and *p* values in the included studies. In some of them, particularly in the fMRI-studies, the outcome assessment method in itself might partly explain this trend, since there is a tradition within this research field not to report effect sizes. Merely reporting clusters of brain activation, where the activation unlikely has occurred by chance (*p* < 0.05), is not enough since it does not say anything about the magnitude of this neural response [38]. Even more importantly, only a minority of the included studies reported results of correlation analyses between changes in neuroplasticity with changes in clinical outcomes. It is unclear why such an association has not been investigated in all studies, and this leads to an uncertainty concerning whether the change in neuroplasticity was really mediated by the physical exercise and related to changes in function or whether other variables, not controlled for in the study, influenced neuroplastic changes.

Most of the included studies adopted a pre- and post-test design, which unfortunately may have reduced the ability to demonstrate neuroplasticity. This is due to the fact that, according to the proposed expansion and renormalization model of the human brain, the initial increase in gray matter volume during training is followed by a selection and renormalization phase in which only the most appropriate circuits remain [39]. With that in mind, it is possible that participants in these studies did have initial morphological brain changes that passed undetected because no tests were conducted during the training period. Interestingly, the one included study exploring changes in brain structure did use a more frequent testing and partially support this theory. Sehm et al. reported changes in the gray matter volume already after two training sessions, whereas no significant changes were detected in the later training phases [34].

Despite the aforementioned methodological flaws of the included studies, it is important to keep in mind that this research area is still in its infancy. To date, the majority of published studies on exercise-induced neuroplasticity in humans with PD are small-scaled. Those articles in this review stating to be pilot studies rarely reported on any feasibility aspects or other factors as recommended by the Consolidated Standards of Reporting Trials: extension to randomized pilot and feasibility trials (CONSORT); [40] nor have any of them to date lead to published full-scale RCT's. Authors also rarely report from which population participants have been recruited, and even more seldom are readers told about recruitment rates.

Regarding generalizability, information is lacking whether the participants are representative of the population from which they were recruited, and whether these participants were easily recruited. An increased transparency regarding feasibility aspects, and implications for a future, definite trial, is needed in order for researchers to improve the quality within this field. This type of research is not only highly complex requiring competence and knowledge covering widespread areas, but it is also dependent on large funding in order to be thoroughly planned and successfully completed. The eagerness within the scientific community to explain improvements in physical performance using neuroplastic markers may have rushed the process. However, with the demand from many journals for clinical trials to have a study protocol registered, along with an increased willingness among journals to publish articles reporting on feasibility aspects will undoubtedly increase the transparency of published trials. This will enable researchers not only to learn from each other's mistakes and advances, but hopefully also encourage comparable reporting of outcome data so that larger meta-analysis can be conducted in the future.

The rigorous and systematic methodological procedure that was used in this review is a considerable strength. We used a wider approach than previous reviewers, covering the majority of methods to explore neuroplasticity in PD as of today. We also focused on long-term adaptations of exercise, as opposed to acute effects, since we believed this to be more interesting from a patient perspective. The findings of this systematic review however need to be seen in light of some limitations. The first is that we used a quality index score instead of a domain-based risk of bias assessment tool. The decision to do so was to assess all included studies using the same instrument. Given the different types of study designs, this narrowed our options. We are however aware that by using a quality index score, we report more so on how well the study was conducted by the investigators, instead of how well the study findings approximate the truth.

## 5. Conclusion

The results of this review suggest that physical exercise may have the ability to induce neuroplasticity in people with PD, but more high-quality studies of RCT design are needed. This field of research is still in its infancy, and upcoming studies should focus on developing a scientifically sound methodology and use transparent reporting. Researchers need to prioritize the assessment of neuroplasticity during initial trial design instead of using subsamples or convenience samples from larger randomized trials.

## Figures and Tables

**Table 1:** Characteristics of included studies, participants, and assessments.

Study characteristics		Participant characteristics						Assessment details	
*Author*, *year*	(1) *Design,* (2) *setting,* (3) *recruitment,* (4) *inclusion critera age/Hoehn & Yahr*	*N* included		Age, mean (SD)		Hoehn & Yahr, mean (SD)		*Neuroplasticity evaluation method*	*Behavioral evaluation*
		*Exp*	*Con*	*Exp*	*Con*	*Exp*	*Con*		
Angelucci et al., [31]	(1) Single-arm clinical trial, (2) inpatient, (3) not reported, (4) no/2-3	9	_	62.8 (6.7)	_	2.1 (0.6)	_	Blood sampling: levels of **BDNF** in serum.	UPDRS II-III, PDQ-39, and 6MWT.
Fontanesi et al., [27]	(1) Single-arm clinical trial, (2) inpatient, (3) not reported, (4) no/no	16	_	71.6 (6.8)	_	2.7 (0.4)	_	Blood sampling: evaluation of **BDNF**-TrkB signaling in lymphocytes.	UPDRS, 6MWT, BBS, TUG, PDDS, FOG-Q.
Frazzitta et al., [28]	(1) Randomized controlled trial, (2) inpatient, (3) recruited from patients admitted to a rehabilitation institute, inclusion, (4) no/1–1.5	15	10	67 (5)	65 (4)	NR	NR	Blood sampling: levels of **BDNF** in serum.	UPDRS III (training group was also tested on UPDRS total, BBS, and 6MWT)
Zoladz et al., [33]	(1) Single-arm clinical trial, (2) outpatient, (3) not reported, (4) no/no	12	_	70 (10.4)^∗^	_	2.3(0.7)^∗^	_	Blood sampling: levels of **BDNF** in serum.	UPDRS total
Batson et al., [35]	(1) Case study, (2) outpatient, (3) recruited from previous intervention group, (4) no/no	1	_	60	_	3	_	Imaging: BOLD **fMRI** signal during rest and with a reaction time task.	None
Duchesne et al., [32]	(1) Single-arm clinical trial, (2) outpatient (3) not reported, (4) no/1–2	19	_	59 (7.11)	_	2 (0)	_	Imaging: BOLD **fMRI** signal during a serial reaction time task.	Cardiovascular fitness evaluated through either a submax- or max-test (bike). Reaction times and accuracy on the serial reaction time task.
Maidan et al., [19]	(1) Randomized controlled trial, (2) outpatient, (3) convenience sample from another project, (4) 60–90/2–3	17	17	71.2 (1.7)	71.5 (1.5)	NR	NR	Imaging: BOLD **fMRI** signal during motor imagery/imagined walking.	Gait speed and stride length during usual walking and obstacle negotiation. Global cognitive function, attention and executive function was assessed using a computerized test battery.
Shah et al., [30]	(1) Randomized controlled trial, (2) outpatient, (3) not reported, (4) 30–65/2–3	13	14	56.5(9.5)	57.2 (7.1)	NR	NR	Imaging: BOLD **fMRI**-signal during a complex bilateral finger tapping task, during a continuous fingertip force tracking task and during rest.	None
Carvalho et al., 2016	(1) Randomized controlled trial, (2) outpatient, (3) recruited from an outpatient rehabilitation department (4) 45–80/1–3	5 (AT) and 8 (ST)	9	64.8 (11.9) (AT) 64.1 (9.9) (ST)	62.1 (11.7)	2.6 (0.5) (AT) 2.1 (0.6) (ST)	2.3 (0.5)	**EEG** mean frequency, electrodes divided into six areas: frontal pole, frontal, central, temporal, parietal and occipital.	UPDRS I-IV. Chair-stand test, arm curl test, 2-minute step test, chair sit and reach test, back scratch test, 8-foot up and go test, 10 m walk test, and BBS.
Fisher et al., [25]	(1) Nonrandomized controlled trial, (2) outpatient, (3) recruited from a movement disorder clinic, (4) no/no	5	7 (LI) and 4 (ZI)	NR	NR	NR	NR	Evaluation of corticomotor excitability using single-pulse **TMS** over the primary motor cortex while monitoring MEPs from the FDI muscle.	UPDRS, 10-meter walk test (both self-selected and as fast as possible) and sit to stand test.
Fisher et al., 2013	(1) Randomized controlled trial, (2) outpatient, (3) subset from ongoing study, (4) no/no	2	2	53.5 (2.1)	56.5 (9.2)	NR	NR	Evaluation of DA-D2R binding potential using **PET** imaging with [18F]fallypride.	Turning, UPDRS III and total.
del Olmo et al., [36]	(1) Single-arm clinical trial, (2) outpatient, (3) not reported, (4) no/no	9	_	61.2(5.2)	_	1.9 (0.5)	_	Evaluation of metabolic brain activity with **PET** using 2-deoxy-2[18F]fluoro-D-glucose	Spatiotemporal gait parameters at preferred speed 30 m. Finger tapping for 30 s.
Sehm et al., 2013	(1) Single-arm clinical trial^a^, (2) outpatient, (3) recruited from an outpatient clinic, (4) no/no	20	_	62.9 (7.1)	_	2.1 (0.4)	_	Evaluation of grey matter volume using structural **MRI**.	BBS; behavioral measure: “Time in target,” i.e, the number of seconds participants were able to keep the platform in a horizontal position.

^∗^SD calculated from SEM. Abbreviations: BDNF: brain-derived neutrophic factor; UPDRS: Unified Parkinson's Disease Rating Scale; PDQ-39: Parkinson's Disease Questionnaire-39; 6MWT: 6-minute walk test; BBS: Berg Balance Scale; TUG: Timed Up and Go; NA: not applicable; NR: not reported; EEG: electroencephalogram; CSP: cortical silent period; 8-FT: 8 Foot Up and Go test; BST: Back Scratch Test; CSRT: Chair Sit and Reach Test; ACT: Arm Curl Test; CST: Chair Stand Test; 2-MST: 2-minute step test; 10mWT: 10-meter walk test; TMS: transcranial magnetic stimulation; PET: positron emission tomography.

**Table 2:** Description of interventions and results.

	Intervention characteristics			Results	
*Author, year*	*Intervention group activity*	*Control group activity*	*Dose*	*Neuroplasticity outcomes*	*Behavioral outcomes*
Angelucci et al., [31]	Motor rehabilitation consisting of 3 sessions per day: (1) exercises to promote flexibility, relaxation, coordination, posture, and walking, (2) treadmill (aerobic) and Wii fit balance board, (3) motor therapy.	NA	4 wks 15 times/wk 60 min	BDNF levels at end comparable to baseline (*p* < 0.14).	Improvements at end: UPDRS II (*p* < 0.05), UPDRS III (*p* < 0.005), PDQ-39 (*p* < 0.01), 6MWT (*p* < 0.05).

Fontanesi et al., [27]	Multidisciplinary rehab 3 sessions per day: (1) physical therapy including ROM, strength, and balance; (2) aerobic training; and (3) occupational therapy.	NA	4 wks 15 times/wk 60 min	Analysis revealed a posttraining upregulation of BDNF-TrkB signaling in the peripheral lymphocytes at the levels of receptors, intracellular mediators, and downstream effectors.	Improvement on all scores: UPDRS total (*p* < 0.01), UPDRS II (*p* < 0.01), UPDRS III (*p* < 0.01), UPDRS IV (*p* = 0.009), 6MWT (p < 0.01), BBS (*p* < 0.01), TUG (*p* = 0.004), PDDS (*p* < 0.01), and FOG-Q (*p* < 0.01).

Frazzitta et al., [28]	Multidisciplinary rehab 3 sessions per day: (1) cardiovascular warm-up, relaxation, stretching, etc.; (2) balance and gait training on platform and treadmill (aerobic training); and (3) occupational therapy.	Passive	4 wks 15 times/wk 60 min	Posttraining analysis revealed increased levels of BDNF in the training group (ES 1.1, *p* < 0.001), while they remained unchanged in the control group (*p* > 0.5).	Participants in the training group improved on UPDRS III compared to controls (*p* ≤ 0.001). Improvement on all other functional outcome measures in the training group (not tested in controls): UPDRS II (*p* ≤ 0.001), UPDRS total (*p* ≤ 0.001), BBS (*p* = 0.002), and 6MWT (*p* ≤ 0.001)

Zoladz et al., [33]	Bike (aerobic) at voluntary-rate warm-up and cooldown 10 min each, and 40 min moderate intensity interval exercise in-between.	NA	8 wks 3 times/wk 60 min	Posttraining analysis of BDNF levels revealed an increase of 34% (*p* = 0.03).	Participants decreased their UPDRS-total score significantly after the training period (*p* = 0.01).

Batson et al., [35]	Improvisational dance emphasizing large ROM, changes in base of support and movement speed variability.	NA	1 wk 5 times/wk 60 min	Stronger connections between anterior and posterior aspects of Default Mode Network. The basal ganglia became highly interconnected with the premotor cortex.	No other outcomes to report.

Duchesne et al., [32]	Bike (aerobic), starting at 20 min and 60% intensity (based on each participant fitness level) and intensified each week until reaching 40 min of training at 80% intensity.	NA	12 wks 3 times/wk 60 min	Analysis revealed a posttraining increase in brain responses in the temporal lobes, left ventral striatum, left hippocampus, cerebellar lobules 8 and 9 bilaterally, and right crus. These responses reflected motor sequence learning capacity specifically.	Improvement in aerobic fitness (V02 max) (*p* < 0.003). Improvement in reaction time for sequential condition only (*p* < 0.006), and also a significant sequence learning effect (*p* < 0.005).

Maidan et al., [19]	Treadmill training. Speed and duration of the treadmill was progressed throughout the training period according to each participant's ability.	Treadmill training with added virtual reality component.	6 wks 3 times/wk 45 min	Posttraining analysis showed significantly different patterns of brain activation between training arms i Brodmann area 10 (*p* = 0.043) and middle temporal gyrus (*p* = 0.005).	Improvements at end: UPDRS II (*p* < 0.05), UPDRS III (*p* < 0.005), PDQ-39 (*p* < 0.01), 6MWT (*p* < 0.05).

Shah et al., [30]	Bike (aerobic) at forced-rate exercise.	Bike (aerobic) at voluntary-rate exercise.	8 wks 3 times/wk 60 min	Posttraining analysis revealed that participants exercising at forced-rate showed that the active motor cortex had a stronger connection to the ipsilateral thalamus compared to those participants who pedaled at voluntary-rate.	No other outcomes to report.

Carvalho et al., [29]	Treadmill training (aerobic) or strength training.	Calisthenics	12 wks 2 times/wk 40 min	EEG analysis showed a higher mean frequency in treadmill and strength training groups compared to calisthenics (*p* = 0.00). No significance regarding brain areas (*p* = 0.97), moment (pre-post) (*p* = 0.89) or any interactions (moment×group (*p* = 0.09), moment×area (*p* = 0.93), group×area (*p* = 0.99), moment×grou*p*×area (*p* = 0.93)).	(Effect size strength training/treadmill/calisthenics) (*p* value): UPDRS I (-0.93, 0.00, −0.21) (*p* = 0.405) UPDRS II (−0.46, −1.12, 0.13) (*p* = 0.313) UPDRS III (−1.25, −1.34, −0.07) (*p* = 0.287) UPDRS IV (−0.46, 1.23, 0.51) (*p* = 0.322) 8-FT (−1.18, −1.08, −1.35) (*p* = 0.859) BST (−0.38, −0.79, −0.41) (*p* = 0.338) CSRT (−0.28, −0.06, 0.08) (*p* = 0.735) ACT (0.74, 1.16, 0.07) (p = 0.271) CST (1.81, 0.86, 0.57) (*p* = 0.328) 2-MST (0.72, 0.69, −0.73) (*p* = 0.012) 10mWT (−0.78, −1.20, −0.34) (*p* = 0.346) BBS (0.44, 0.38, 0.00) (*p* = 0.721)

Fisher et al., 2008	High-intensity body weight-supported treadmill training (aerobic).	Low intensity physical therapy (active and passive ROM training, balance, gait, resistance and functional training) or passive control group.	8wks 3 times/wk 60 min	All subjects in the treadmill group showed an increased CSP-duration in both hemispheres after training. No changes were found in the physical therapy group.	Not reported for the TMS subsample.

Fisher et al., 2013	Treadmill (aerobic), each session aimed at reaching and maintaining a metabolic equivalent of task level greater than 75% of age-adjusted heart rate.	Passive	8 wks 3 times/wk 60 min	PET imaging post-training demonstrated a marked increase in Fallypride BP in the dorsal putamen in both individuals. No changes were seen in control subjects.	Exercise subjects demonstrated improved turning performance, while control subjects did not. No participant improved in either UPDRS total or UPDRS III.

del Olmo et al., [36]	Gait and fingertapping training with and without rhythmic auditory cues.	NA	4 wks 5 times/wk 60 min	PET imaging post-training revealed a metabolic increment in the right cerebellum (*p* < 0.001) and in the right parietal and temporal lobes (*p* < 0.001).	Coefficient of variation decrement for both fingertapping (*p* < 0.05) and gait (*p* < 0.01). No significant change on all other parameters.

Sehm et al., 2013	Balance training using a movable platform.	NA	6 wks 1 time/wk 45 min	Imaging analysis revealed a post-training increase in grey matter volume in the right hemisphere of the cerebellum (lobule V–VI) (*p* < 0.05).	Participants showed a significant increase in DBT (time in target). Results on BBS are not reported.

Abbreviations: Exp—Experimental group, Con—Control group, BDNF—Brain-derived Neutrophic Factor, UPDRS—Unified Parkinson's Disease Rating Scale, PDQ-39—Parkinson's Disease Questionnaire-39, 6MWT—6-minute walk test, BBS—Berg Balance Scale, TUG—Timed Up and Go, AT—Aerobic training, ST—Strength training, LI—Low intensity, ZI—Zero intensity, NR—Not reported, EEG—Electroencephalogram, CSP—Cortical Silent Period, 8-FT—8 Foot Up and Go test, BST—Back Scratch Test, CSRT—Chair Sit and Reach Test, ACT—Arm Curl Test, CST—Chair Stand Test, 2-MST—2 Minute Step Test, 10mWT—10 meter Walk Test, TMS—Transcranial Magnetic Stimulation, PET—Positron Emission Tomography, BP—Binding Potential.

**Table 3:** Narrative synthesis of neuroplastic outcomes from pre- to post-intervention.

Reference	Quality	Method used to measure neuroplasticity	Specification of signaling type or brain area/s		Direction of change from pre to post-intervention	MA^∗^
Angelucci et al.	13	Blood sampling, BDNF serum concentration			0	Yes
Fontanesi et al.	14	Blood sampling, TrkB signaling in lymphocytes	pY-TrkB (145 kDa)		+	No
			pY-TrkB (95 kDa)		0	No
			NR1		+	No
**Frazzitta et al.**	20	Blood sampling, BDNF serum concentration			+	Yes
Zoladz et al.	15	Blood sampling, BDNF serum concentration			+	Yes

				**Activity change from pre to post-intervention**		
Batson et al.	6	fMRI, BOLD signal	Connection between anterior and posterior aspects of Default Mode Network	+	+	No
			Connection between basal ganglia and premotor cortex	+	+	No
Duchesne et al.	14	fMRI, BOLD signal	Temporal lobes	+	+	No
			Left ventral striatum	+	+	No
			Left hippocampus	+	+	No
			Cerebellum (lobules 8 and 9 bilaterally and right crus)	+	+	No
**Maidan et al.**	15	fMRI, BOLD signal	Middle temporal gyrus	−	+	No
Shah et al.	14	fMRI, BOLD signal	Connection between active motor cortex and ipsilateral thalamus	+	+	No
**Carvalho et al.**	16	EEG, mean frequency	No significance given to area	+	+	No
**Fisher et al.** [25]	14	TMS, CSP duration	CSP-duration in both hemispheres	+	+	No
**Fisher et al. (2013)**	16	PET, metabolic activity	Dorsal putamen	+	+	No
del Olmo et al.	13	PET, metabolic activity	Right cerebellum	+	+	No
			Right parietal lobe	+	+	No
			Right temporal lobe	+	+	No

				**Volume change from pre to post-intervention**		
Sehm et al.	15	MRI	Right hemisphere of cerebellum^∗∗^	+	+	No

References in bold are of RCT design. Overall quality score assessed with the modified Down and Black checklist, ranging from 0 to 28 with higher scores indicating higher overall quality. Activity and volume indicate whether a change in respective measure increased (+) or decreased (−). Direction indicates change in neuroplastic marker from pre to post-intervention and states whether it was assessed as positive (+), negative (−), or unchanged (0). ∗ indicates whether the study was included in the meta-analysis (MA). ∗∗ as compared to healthy control group. Abbreviations: BDNF—Brain Derived Neurotrophic Factor; TrkB—Tyrosine receptor kinase B; fMRI—Functional Magnetic Resonance Imaging; BOLD—Blood Oxygenated Level Dependent; EEG—Electroencephalogram; TMS—Transcranial Magnetic Stimulation; CSP—Cortical Silent Period; PET—Positron Emission Tomography.

**Table 4:** Summary of findings and overall level of evidence as assessed with GRADE.

		All studies	Studies included in the meta-analysis			GRADE domains					
Domain	Method	Total number of participants (no. studies)	Total number of participants (no. studies)	Estimated effect size (*p* value)	Phase	Study limitations	Inconsistency	Indirectness	Imprecision	Publication bias	Level of evidence
Neurochemical	BDNF level in serum, TrkB signaling in lymphocytes	61 (4)	36 (3)	0.91–1.84 (>0.05)	+++	−	0^∗^	0	−	0^∗∗^	+ Very low
Brain function	fMRI, EEG, TMS, PET	132 (8)	NA	NA							
Brain structure	MRI	20 (1)	NA	NA							

GRADE—Grading of Recommendations Assessment, Development and Evaluation. ^∗^Heterogenous outcomes. ^∗∗^None of the included studies were registered in Clinical Trials.
