# Supplementary material for: Exercise-Induced Neuroplasticity in Parkinson's Disease: A Metasynthesis of the Literature
Source: Neural Plast. 2020 Mar 5;2020:8961493. doi: 10.1155/2020/8961493 (PMC7079218; doi:10.1155/2020/8961493)
Supplement: Supplementary Materials — The following Supplementary Materials are available for this paper: search strategy and excluded articles with reasons and quality assessment. [file 8961493.f1.docx]

**Supplementary Material.**

Search strategy

1. Parkinson Disease/

2. parkinson*.ti,ab,kf.

3. or/1-2
4. exp Exercise/

5. exp Exercise therapy/
6. Physical Therapy Modalities/
7. Equine-assisted therapy/
8. exp Exercise Movement Techniques/
9. Dancing/
10. Sports/
11. Baseball/
12. Basketball/
13. Bicycling/
14. Boxing/
15. Football/
16. Golf/
17. Gymnastics/
18. Hockey/
19. exp Martial Arts/
20. Mountaineering/
21. exp Racquet Sports/
22. exp Running/
23. Skating/
24. exp Snow Sports/
25. Soccer/
26. Sports for Persons with Disabilities/
27. exp Swimming/
28. "Track and Field"/
29. Volleyball/
30. Weight Lifting/
31. Wrestling/
32. (aerobic* or aikido or archery or athletics or badminton or ballgame* or ball game* or baseball or basketball or biathl* or bicycle* or bicycling or bowling or boxing or calisthenic* or cardiopulmonary conditioning or callisthenic* or canoe* or cricket or crossfit or curling or cycling or danc* or diving or exercise or fencing or floorball or floor ball or football or golf* or jogging or jumping or handball or hopping or gigong or gi gong or gymnastic* or hiit or hockey or horseback riding or horse riding or isometric climbing or judo or jujitsu or karate or kung fu or kung or marathon* or martial art* or mountaineer* or multidisciplinary rehabilitation or neuromuscular facilitation* or orienteering or physical acitivit* or power lifting or pilates or plyometric* or qi gong or qigong or racquetball or racketball or racket ball or rowing or rugby or running or sailing or skateboard* or skating or skiing or snowboard* or soccer or softball or squash or sport* or stretching or swim* or tae kwon do or taekwondo or tai or thai or taiji or taijiquan or taichi or tango or tennis or treadmill or walk* or warm* up or warmup* or volleyball or weight* lifting or lifting weight* or weightlifting or workout* or work out* or wrestling or yoga or zumba).ti,ab,kf.
33. ((equine or motion or movement) adj2 therap*).ti,ab,kf.

34. ((balance or circuit or concentric* or conditioning or cool* down or cooldown or eccentric*or fitness or isometric* or physical or motion or movement or muscle* or strength or resistance or warm* down) adj2 (exercise* or program* or train*)).ti,ab,kf.
35. or/4-34
36. exp Nerve growth factors/
37. exp Neuronal Plasticity/
38. exp Tomography/
39. exp Proteins/
40. exp Diagnostic Techniques, Neurological/
41. exp Biomarkers/
42. Transcranial Magnetic Stimulation/
43. Nerve Degeneration/
44. exp Brain/
45. exp Blood/
46. blood.fs.
47. exp Cerebrospinal Fluid/
48. cerebrospinal fluid.fs.
49. (amyloid beta or biomarker* or blood* or bdnf or cerebrospinal fluid* or chemical shift imag* or csf or d2 or diffusion tensor imag* or dopamine or electroencephalograph* or fmri or magnetic resonance* or magnetization transfer contrast imag* or magnetoencephalograph* or meg or mr or mri or nerve growth factor* or neuroendoscop* or neuroimag* or neuronal protect* or neuroplastic* or neuroprotect* or neurotroph* or peptide* or pet or plasticit* or protein* or receptor* or tomograph* or transmitter* or spinal fluid* or synuclein* or tau or tms or transcranial magnetic stimulation).ti,ab,kf.

50. ((nerve* or neurons or neuron or neuronal*) adj2 (imaging or degenerat*)).ti,ab,kf. 51. (brain or cerebell* cerebral or epithalam* or hippocamp*).ti,ab,kf.
52. or/36-51
53. 3 and 35 and 52

54. remove duplicates from 53 55. exp animals/
56. humans/
57. 55 not 56

58. 54 not 57

**Supplementary Material.**

Excluded articles

| **Title** | **Author** | **Year** | **Reason for exclusion** |
| --- | --- | --- | --- |
| Brain plasticity in Parkinson's disease with freezing of gait induced by action observation training | Agosta et al | 2017 | Intervention primarily focused on observation, not physical exercise. |
| It is not about the bike, it is about the pedaling: forced exercise and Parkinson's disease | Alberts et al | 2011 | Review |
| Physical exercise and depression | ann het Rot et al | 2009 | Review (not on Parkinson´s Disease) |
| Exercise: A workout for neuroregeneration | Azizi et al | 2007 | Commentary - Not original paper |
| The effect of forced-exercise therapy for Parkinson's disease on motor cortex functional connectivity | Beall et al | 2013 | One exercise session only |
| Effect of resistance training on blood oxidative stress in Parkinson disease | Bloomer et al | 2008 | No neuroplasticity outcome |
| On the move to stimulate cell plasticity in the substantia nigra in Parkinson's disease | Brundin et al | 2006 | Commentary - Not original paper |
| Cyclic exercise induces anti-inflammatory signal molecule increases in the plasma of Parkinson's patients | Cadet et al | 2003 | No neuroplasticity outcome |
| A two-year randomized controlled trial of progressive resistance exercise for Parkinson's disease | Corcos et al | 2013 | No neuroplasticity outcome |
| Controlled pilot study of the effects of neuromuscular therapy in patients with Parkinson's disease | Craig et al | 2006 | No neuroplasticity outcome |
| Parkinson's patient runs an ultra marathon: a case report | Daviet et al | 2014 | One exercise session only (marathon) |
| Exercise and Medication Effects on Persons With Parkinson Disease Across the Domains of Disability: A Randomized Clinical Trial | Dibble et al | 2015 | No neuroplasticity outcome |
| Neurophysiological adaptations to resistance training and repetitive grasping | Falvo | 2010 | Thesis |
| Parkinson's disease rehabilitation: A pilot study with 1 year follow up | Frazzitta et al | 2010 | No neuroplasticity outcome |
| Another reason to exercise | Gitler | 2011 | Perspective - Not original paper |
| The Healing Power of Music and Dance | Horowitz | 2013 | Review |
| No evidence of neuroprotection? Perhaps not...perhaps so | Lewin et al | 2014 | Correspondence - Not original paper |
| Levodopa normalizes exercise related cortico-motoneuron excitability abnormalities in Parkinson's disease | Lou et al | 2003 | One exercise session only |
| Repetitive transcranial magnetic stimulation combined with treadmill training can modulate corticomotor inhibition and improve walking performance in people with Parkinson's disease | Mak et al | 2013 | Commentary - Not original paper |
| Evaluating rehabilitation interventions in Parkinson’s disease with functional MRI: A promising neuroprotective strategy | Mallio et al | 2015 | Perspective - Not original paper |
| Interval training-induced alleviation of rigidity and hypertonia in patients with Parkinson´s disease is accompanied by increased basal serum brain-derived neurotrophic factor. | Marusiak et al | 2015 | Same studied population and neuroplasticity outcome as other included study (Zoladz et al, 2014) |
| Aerobic exercise combined with rTMS for Parkinson's disease: A randomized trial | Migdadi et al | 2017 | No physical exercise intervention |
| Therapy of patients with Parkinson's disease | Paulson | 1994 | Editorial - Not original paper |
| Mindfulness based intervention in Parkinson's disease leads to structural brain changes on MRI: a randomized controlled longitudinal trial | Pickut et al | 2013 | Review |
| Two-year exercise program improves physical function in Parkinson's disease: the PRET-PD randomized clinical trial | Prodoehl et al | 2015 | No neuroplasticity outcome |
| Physical exercise in Parkinson disease: Moving toward more robust evidence? | Rascol | 2013 | Editorial |
| Both the body and brain benefit from exercise: potential win-win for Parkinson's disease patients | Weintraub et al | 2011 | Hot topics, review of other article |
| Combination of rTMS and treadmill training modulates corticomotor inhibition and improves walking in Parkinson disease: a randomized trial | Yang et al | 2013 | Mixed intervention (rTMS and treadmill) |

**Supplementary Material**

Quality scoring according to Downs and Black

| **Author** | **Reporting** | | | | | | | | | | **External validity** | | | **Internal validity - bias** | | | | | | | **Internal validity - confounding** | | | | | | **Power** | **Quality score** |
| --- | --- | --- | --- | --- | --- | --- | --- | --- | --- | --- | --- | --- | --- | --- | --- | --- | --- | --- | --- | --- | --- | --- | --- | --- | --- | --- | --- | --- |
|  | ***1*** | ***2*** | ***3*** | ***4*** | ***5*** | ***6*** | ***7*** | ***8*** | ***9*** | ***10*** | ***11*** | ***12*** | ***13*** | ***14*** | ***15*** | ***16*** | ***17*** | ***18*** | ***19*** | ***20*** | ***21*** | ***22*** | ***23*** | ***24*** | ***25*** | ***26*** | ***27*** |  |
| Angelucci et al | 1 | 1 | 1 | 1 | 1 | 1 | 0 | 0 | 1 | 0 | 0 | 0 | 0 | 0 | 0 | 1 | 1 | 1 | 1 | 1 | 0 | 0 | 0 | 0 | 0 | 1 | 0 | 13 |
| Batson et al | 1 | 1 | 0 | 0 | 0 | 0 | 0 | 0 | 1 | 0 | 0 | 0 | 0 | 0 | 0 | 0 | 0 | 1 | 1 | 0 | 0 | 0 | 0 | 0 | 0 | 1 | 0 | 6 |
| Carvalho et al | 1 | 1 | 1 | 1 | 1 | 0 | 1 | 0 | 1 | 0 | 0 | 0 | 0 | 0 | 1 | 1 | 1 | 1 | 1 | 1 | 1 | 0 | 1 | 0 | 0 | 1 | 0 | 16 |
| Duchesne et a | 1 | 1 | 1 | 1 | 1 | 1 | 0 | 0 | 1 | 1 | 0 | 0 | 0 | 0 | 0 | 1 | 1 | 1 | 1 | 1 | 0 | 0 | 0 | 0 | 0 | 1 | 0 | 14 |
| Fisher et al | 1 | 1 | 1 | 1 | 0 | 1 | 1 | 1 | 1 | 0 | 0 | 0 | 0 | 0 | 1 | 1 | 1 | 1 | 1 | 1 | 0 | 0 | 1 | 0 | 0 | 1 | 0 | 16 |
| Fisher et al | 1 | 1 | 0 | 1 | 0 | 1 | 1 | 0 | 1 | 0 | 0 | 0 | 0 | 0 | 1 | 1 | 1 | 1 | 1 | 1 | 0 | 0 | 1 | 0 | 0 | 1 | 0 | 14 |
| Fontanesi et a | 0 | 1 | 1 | 1 | 1 | 1 | 1 | 0 | 1 | 0 | 0 | 0 | 0 | 0 | 1 | 1 | 1 | 1 | 1 | 1 | 0 | 0 | 0 | 0 | 0 | 1 | 0 | 14 |
| Frazzitta et al | 1 | 1 | 1 | 1 | 1 | 1 | 1 | 0 | 1 | 1 | 0 | 0 | 0 | 0 | 1 | 1 | 1 | 1 | 1 | 1 | 1 | 1 | 1 | 0 | 0 | 1 | 1 | 20 |
| Maidan et al | 1 | 1 | 1 | 1 | 1 | 1 | 0 | 0 | 1 | 1 | 0 | 0 | 0 | 0 | 1 | 1 | 1 | 1 | 0 | 1 | 1 | 0 | 0 | 0 | 1 | 0 | 0 | 15 |
| Del Olmo et al | 1 | 1 | 1 | 1 | 1 | 1 | 1 | 0 | 1 | 0 | 0 | 0 | 0 | 0 | 0 | 1 | 1 | 1 | 1 | 1 | 0 | 0 | 0 | 0 | 0 | 0 | 0 | 13 |
| Sehm et al | 1 | 1 | 1 | 1 | 1 | 1 | 1 | 0 | 1 | 1 | 0 | 0 | 0 | 0 | 0 | 1 | 1 | 1 | 1 | 0 | 1 | 0 | 0 | 0 | 0 | 1 | 0 | 15 |
| Shah et al | 1 | 1 | 1 | 1 | 1 | 1 | 0 | 0 | 1 | 0 | 0 | 0 | 0 | 0 | 0 | 1 | 1 | 1 | 1 | 1 | 0 | 0 | 1 | 0 | 0 | 1 | 0 | 14 |
| Zoladz et al | 1 | 1 | 1 | 1 | 1 | 1 | 1 | 0 | 1 | 1 | 0 | 0 | 0 | 0 | 1 | 0 | 1 | 1 | 1 | 1 | 0 | 0 | 0 | 0 | 0 | 1 | 0 | 15 |
